# UBE3C Facilitates the ER-Associated and Peripheral Degradation of Misfolded CFTR

**DOI:** 10.3390/cells12232741

**Published:** 2023-11-30

**Authors:** Yuka Kamada, Hazuki Tateishi, Uta Nakayamada, Daichi Hinata, Ayuka Iwasaki, Jingxin Zhu, Ryosuke Fukuda, Tsukasa Okiyoneda

**Affiliations:** Department of Biomedical Sciences, School of Biological and Environmental Sciences, Kwansei Gakuin University, Hyogo 669-1330, Japan; yuka.kamada@kwansei.ac.jp (Y.K.); gtn09441@kwansei.ac.jp (H.T.); hvn03445@kwansei.ac.jp (U.N.); daichi.hinata@kwansei.ac.jp (D.H.); gst59800@kwansei.ac.jp (A.I.); gps59816@kwansei.ac.jp (J.Z.); r.fukuda@kwansei.ac.jp (R.F.)

**Keywords:** UBE3C, CFTR, ABCB1, protein quality control, ERAD, ubiquitin, RNF185, RNF5

## Abstract

The ubiquitin E3 ligase UBE3C promotes the proteasomal degradation of cytosolic proteins and endoplasmic reticulum (ER) membrane proteins. UBE3C is proposed to function downstream of the RNF185/MBRL ER-associated degradation (ERAD) branch, contributing to the ERAD of select membrane proteins. Here, we report that UBE3C facilitates the ERAD of misfolded CFTR, even in the absence of both RNF185 and its functional ortholog RNF5 (RNF5/185). Unlike RNF5/185, UBE3C had a limited impact on the ubiquitination of misfolded CFTR. UBE3C knockdown (KD) resulted in an additional increase in the functional ∆F508-CFTR channels on the plasma membrane when combined with the RNF5/185 ablation, particularly in the presence of clinically used CFTR modulators. Interestingly, although UBE3C KD failed to attenuate the ERAD of insig-1, it reduced the ERAD of misfolded ∆Y490-ABCB1 and increased cell surface expression. UBE3C KD also stabilized the mature form of ∆F508-CFTR and increased the cell surface level of T70-CFTR, a class VI CFTR mutant. These results suggest that UBE3C plays a vital role in the ERAD of misfolded CFTR and ABCB1, even within the RNF5/185-independent ERAD pathway, and it may also be involved in maintaining the peripheral quality control of CFTR.

## 1. Introduction

Cystic fibrosis (CF) transmembrane conductance regulator (CFTR) is a cAMP-regulated chloride (Cl^−^) channel expressed at the apical plasma membrane (PM) of epithelial cells [1]. Mutations in the CFTR gene lead to cystic fibrosis (CF), which stands as one of the most prevalent inherited diseases among individuals of Caucasian descent [1,2,3]. The most common mutant in CF patients is ∆F508-CFTR, in which phenylalanine at position 508 in the cytosolic nucleotide-binding domain 1 (NBD1) is deleted [4,5]. The ∆F508 mutation results in CFTR conformational instabilities, including the NBD1 and the domain interface between the NBD1 and membrane-spanning domains (MSD1 and MSD2) [6,7,8]. Consequently, ∆F508-CFTR fails to properly attain the native conformation at the ER, resulting in premature degradation through the ubiquitin (Ub)-proteasome system, known as ER-associated degradation (ERAD) [9,10]. Several Ub E3 ligases are involved in the CFTR ERAD. A cytosolic chaperone-associated E3 ligase CHIP/STUB1 recognizes the cytoplasmic regions of CFTR, such as the unstable NBD1, and facilitates CFTR ubiquitination at the late CFTR biosynthesis stage [11]. In parallel, the ER-embedded E3 ligase RNF5 [12] and its paralog RNF185 [13] are involved in the misfolded CFTR ubiquitination at the early biosynthesis stage. Furthermore, Gp78/AMFR acts as an E4 ligase to extend the poly-Ub chains on misfolded CFTR initiated by RNF5 [14]. Recently, it has been shown that RNF185 forms an ERAD complex along with membralin (MBRL/TMEM259) and TMUB1/2. This ERAD complex plays a crucial role in removing misfolded membrane proteins, including CYP51A1 [15]. Moreover, it has been suggested that cytosolic E3 UBE3C works downstream of the RNF185/MBRL ERAD branch [15]. The yeast UBE3C ortholog Hul5 is believed to function on the proteasome as an E4 enzyme that elongates Ub chains on the proteasome-bound substrates, thereby increasing their degradation [16]. UBE3C also appears to enhance the degradation of misfolded cytosolic proteins, especially upon heat shock [17].

While most ∆F508-CFTR is eliminated by the ERAD pathway, it is also eliminated by the peripheral quality control (QC) mechanism when reaching the PM [18,19]. Cell surface ∆F508-CFTR, which is increased at low temperatures [20] and/or therapeutic agents such as CFTR correctors [21], still has conformational defects, thereby eliminated by Ub-dependent endo-lysosomal degradation [18,19]. The conformationally defective ∆F508-CFTR at the PM is ubiquitinated by CHIP [19], RFFL [22], and RNF34 [23]. Inhibition of these E3 ligases increases the functional ∆F508-CFTR channel at the PM by preventing rapid internalization and lysosomal degradation [19,22]. The unidentified other E3 ligases may also participate in the CFTR peripheral QC.

In this study, we demonstrate that UBE3C promotes the ERAD of misfolded CFTR and ABCB1, even when both RNF185 and its functional ortholog RNF5 are absent. We employed a recently established HiBiT degradation assay [24], along with knockdown (KD) experiments, to illustrate that UBE3C KD delays the ERAD of misfolded ∆F508-CFTR, N1303K-CFTR, and ∆Y490-ABCB1. Importantly, these effects were also observed in cells in which both RNF5 and RNF185 had been ablated. Interestingly, unlike RNF5 and RNF185, both of which contribute to the ubiquitination of CFTR, UBE3C had a minimal impact on the ubiquitination of ∆F508-CFTR. The UBE3C KD resulted in an increase in the pool of ∆F508-CFTR and ∆Y490-ABCB1 that can be properly folded within the ER. This, in turn, led to a higher cell surface expression of these misfolded ABC transporters when their folding correctors were present. These results indicate that UBE3C plays a critical role in the ERAD of misfolded membrane proteins such as CFTR and ABCB1 through the RNF5/185-independent ERAD mechanism. Additionally, our findings suggest that UBE3C may be involved in maintaining the peripheral QC of CFTR.

## 2. Materials and Methods

### 2.1. Reagents and Antibodies

The following chemicals were used: DMSO (Sigma-Aldrich, St Louis, MO, USA, Cat# D2650), MG-132 (Cayman Chemical, Ann Arbor, MI, USA, Cat# 10012628), VX-661 (Selleck Chemicals, Houston, TX, USA, Cat# S7059), VX-445 (Selleck Chemicals, Cat# S8851), VX-770 (Chemscene LLC, Monmouth Junction, NJ, USA, Cat# CS-0497), cycloheximide (CHX, FUJIFILM Wako Pure Chemical Corporation, Osaka, Japan, Cat# 3720991), doxycycline (Dox, FUJIFILM Wako Pure Chemical Corporation, Cat# 049-31121), cyclosporin A (CLP-A, FUJIFILM Wako Pure Chemical Corporation, Cat# 031-24931).

The following antibodies were used: mouse anti-HA (16B12, BioLegend, San Diego, CA, USA, Cat# 901515), anti-K48-Ub (Apu2 ZooMAb, Sigma-Aldrich, Cat# ZRB2150), anti-UBE3C (Abcepta, Inc., San Diego, CA, USA, Cat# AP20457c), anti-GST (clone 5A7, FUJIFILM Wako Pure Chemical Corporation, Cat# 013-21851), anti-Ub (P4D1, Santa Cruz Biotechnology, Santa Cruz, CA, USA, Cat# sc-8017), anti-DYKDDDDK (anti-FLAG, clone 1E6, FUJIFILM Wako Pure Chemical Corporation, Cat# 014-22383), anti-Myc (clone 9E10, JIFILM Wako Pure Chemical Corporation, Cat# 017-21871), RNF185 antiserum (anti-RNF185) from rabbit [25], anti-HiBiT (Promega, Madison, WI, USA, Cat# N7200), Peroxidase AffiniPure Goat Anti-Mouse IgG (H+L) Secondary Antibody (Jackson Immuno Research, West Grove, PA, USA, Cat# 115–035-166), Peroxidase AffiniPure Donkey Anti-Rabbit IgG (H+L) Secondary Antibody (Jackson Immuno Research, Cat# 711–035-152).

### 2.2. Plasmids

∆F508-CFTR-HiBiT(CT), N1303K-CFTR-HiBiT(CT), Insig-1-HiBiT(CT), ∆Y490-ABCB1-HiBiT(CT), and ∆F508-CFTR-Nluc(Ex) were constructed previously [23,24,25]. pCold-GST-4xTR-TUBE (GST-TUBE) was constructed by in-fusion cloning (Takara Bio, Kusatsu, Japan) using the pCold-GST plasmid [25] and pRSET-4xGST-TR-TUBE (Addgene #110312) as templates. pNUT-∆F508-CFTR-3HA [19] and pCold-His-sumo-USP21 (196–565) were kindly provided by Dr. Gergely Lukacs (McGill University) and Dr. Yusuke Sato (Tottori University), respectively. T70-CFTR-HiBiT(Ex) was constructed by replacing the 3xHA tag with the HiBiT tag in the 4th extracellular loop of CFTR as previously [25]. ∆Y490-ABCB1-HiBiT(Ex) was constructed by inserting the HiBiT tag in the 1st extracellular loop by PCR-based mutagenesis and inserted to pLX304 (Addgene #25890) via LR reaction using LR clonase II enzyme mix (ThermoFisher Scientific, Waltham, MA, USA). pcDNA3-FLAG-His-UBE3C was constructed by inserting UBE3C cDNA into the pcDNA3.2 vector (ThermoFisher) by in-fusion cloning. The C1051A-UBE3C mutant was generated by PCR-based mutagenesis. pcDNA3-Myc-Ub was constructed by inserting human ubiquitin C cDNA into the pcDNA3.1 vector (ThermoFisher) by ligation. All constructs were verified by DNA sequencing.

### 2.3. Cell Lines and Cell Culture

All the cells used in this study were cultured in 5% CO_2_ at 37 °C. 293MSR (ThermoFisher Scientific, Cat# R79507) and 293MSR-RNF5/185 DKO [24] cells were grown in Dulbecco’s Modified Eagle Medium (DMEM) (FUJIFILM Wako Pure Chemical Corporation) supplemented with 10% fetal bovine serum (FBS). 293MSR or 293MSR-RNF5/185 DKO cells stably expressing HBH-∆F508-CFTR-3HA were grown in DMEM supplemented with 10% FBS, 0.5 mg/mL G418, and 5 µg/mL blasticidin S as previously [24]. CFBE41o-Tet-on cells stably expressing ΔF508-CFTR-3HA and constitutive YFP-H148Q/I152L/F46 (obtained from Dr. Gergely Lukacs, McGill University) were grown in minimal essential medium (MEM, FUJIFILM Wako Pure Chemical Corporation) supplemented with 10% FBS, 2 mM L-glutamine, 10 mM HEPES, and 3 µg/mL puromycin (Sigma-Aldrich), as previously mentioned [22]. For propagation, the CFBE cells were cultured in plastic dishes coated with an extracellular matrix (ECM mix) consisting of 10 mg/mL human fibronectin, 30 mg/mL PureCol collagen preparation (Advanced Biomatrix, Carlsbad, CA, USA), and 100 mg/mL bovine serum albumin (Sigma-Aldrich). BHK and BHK cells stably expressing HBH-∆F508-CFTR were cultured as previously [22]. BEAS-2B cells stably expressing ∆F508-CFTR-Nluc(Ex) [25], ∆F508-CFTR-Nluc(CT) [24], T70-CFTR-HiBiT(Ex), or ∆Y490-ABCB1-HiBiT(Ex) were grown in DMEM supplemented with 10% FBS, and 10 µg/mL blasticidin S. The parental BEAS-2B cells (Cat# 95102433) were obtained from the European Collection of Authenticated Cell Cultures (ECACC). Penicillin-streptomycin solution (FUJIFILM Wako Pure Chemical Corporation) was added to all cell culture mediums. For the Tet-on cells, cells were treated with 1 mg/mL Dox for 2 days to induce CFTR expression.

### 2.4. Transfection

Transient expression of plasmids in 293MSR was accomplished using polyethylenimine Max (Polysciences Inc., Warrington, PA, USA). siRNA transfection in 293MSR, CFBE, and BEAS-2B cells was accomplished using Lipofectamine RNAiMax transfection reagent (ThermoFisher). When not indicated differently, siRNA-transfected cells were used for the experiments 4 days post-transfection. The following siRNAs were used; UBE3A (siUBE3 #1: hs.Ri.UBE3C.13.1, siUBE3 #3: hs.Ri.UBE3C.13.3, Integrated DNA Technologies (IDT), Coralville, IA). As a negative control for siRNA from IDT, dsiNC (siNC, Integrated DNA Technologies) was used. If not specified, siUBE3C #1 was used for UBE3C KD. For RNF5 and RNF185 double KD, the pooled siRNF5 and siRNF185 were used. The pooled siRNA was created by combining an equal quantity of individual siRNAs targeting RNF5 (siRNF5 #11, #6, #7, Qiagen, Hilden, Germany) and RNF185 (siRNF185 #2, #3, #8, Qiagen). AllStars Negative Control siRNA (Qiagen) was also utilized as a negative control for siRNA (siNT) from Qiagen.

### 2.5. PM Density Measurement of CFTR

The PM level of ∆F508-CFTR-Nluc(Ex), T70-CFTR-HiBiT(Ex), or ∆Y490-ABCB1-HiBiT(Ex) in 293MSR and BEAS-2B cells on 96 well plates was measured using the NanoGlo Extracellular Nluc Substrate (Promega, Cat# CS313501) or Nano Glo HiBiT Extracellular system (Promega, Cat# N2421), according to the manufacturer’s instructions as previously [23,24,25]. The luminescent signal was measured using the Varioskan Flash (ThermoFisher) and EnSpire Alpha plate reader (PerkinElmer, Waltham, MA, USA). To induce the cell surface expression of ∆F508-CFTR-Nluc(Ex) and ∆Y490-ABCB1-HiBiT(Ex), cells were treated with Trikafta (3 µM VX-661, 1 µM VX-445, 1 µM VX-770) and 10 µM cyclosporin A (CLP-A, FUJIFILM Wako Pure Chemical Corporation, Cat# 031-24931), respectively, for 24 h at 37 °C.

### 2.6. Quantitative Real-Time PCR

Total RNA was extracted from cells two days post-transfection of siRNA using TRIzol^®^ (ThermoFisher) according to the manufacturer’s protocols. An amount of 500 ng of total RNA was then used for the reverse transcription (RT) reaction using ReverTra Ace^®^ qPCR RT Master Mix (Toyobo, Japan). Quantitative RT-PCR was performed as previously [24]. The relative quantity of the target gene mRNA was normalized using human GAPDH as the internal control. The sequences of primers used for quantitative RT-PCR are listed in Table 1.

### 2.7. Western Blotting

Cells were solubilized in a RIPA buffer supplemented with 1 mM PMSF (FUJIFILM Wako Pure Chemical Corporation), 5 µg/mL leupeptin (FUJIFILM Wako Pure Chemical Corporation), and 5 µg/mL pepstatin A (Peptide Institute Inc., Osaka, Japan), where the cell lysates were analyzed by a Western blot as done previously [22]. Western blots were visualized using a SuperSignal West Pico PLUS Chemiluminescent Substrate (ThermoFisher Scientific), ImmunoStar Zeta (FUJIFILM Wako Pure Chemical Corporation), or ImmunoStar LD (FUJIFILM Wako Pure Chemical Corporation) and analyzed by FUSION Chemiluminescence Imaging System (Vilber BioImaging, Paris, France). The staining of Ponceau S (Sigma-Aldrich) was used as a loading control.

### 2.8. Halide-Sensitive YFP Quenching Assay

∆F508-CFTR function assay by halide-sensitive YFP fluorescence quenching was performed as described [22,24,25,26]. CFBE cells expressing both inducible ∆F508-CFTR-3HA and halide sensor YFP-F46L/H148Q/I152L were seeded onto black 96-well microplates and transfected with siRNA (50 nM each) and dsiRNA (25 nM). Cells were treated with 1 µg/mL Dox for 4 days to induce the CFTR. Cell surface expression of ∆F508-CFTR was induced by Trikafta treatment (3 µM VX-661, 1 µM VX-445, 1 µM VX-770) for 2 days at 37 °C. CFTR inhibitor 172 (20 µM, CFTRinh-172, Selleck Chemicals, Cat# S7139) was pre-treated before cAMP activation as previously [25]. Fluorescence measurements were conducted using a VICTOR Nivo multimode microplate reader (Perkin Elmer) equipped with a dual syringe pump (excitation/emission 500/535 nm). After normalizing the YFP signals before PBS-iodide injection, the rate of iodide (I^−^) influx was determined by fitting the YFP fluorescence decay curve using GraphPad Prism 8 software (GraphPad Software).

### 2.9. HiBiT Degradation Assay

The HiBiT degradation assay was performed as previously [24]. Briefly, 293MSR and RNF5/185 DKO cells in 6-well plates were subjected to siRNA treatment (25 nM). Following one day of incubation, the cells were detached, transferred to new 6-well plates, and allowed to grow for an additional day. Afterward, the cells underwent transfection with ∆F508-CFTR-HiBiT(CT), Insig-1-HiBiT(CT), N1303K-CFTR-HiBiT(CT), or ∆Y490-ABCB1-HiBiT(CT) along with cytosolic LgBiT (pBiT1.1-N [TK/LgBiT], Promega, Madison, WI, USA).

The following day, the cells were detached, seeded in a 96-well plate, and cultured for 18–24 h. After loading Nano-Glo^®^ Endurazine (Promega), luminescence was regularly recorded at 5-min intervals utilizing a Luminoskan plate reader (ThermoFisher). The luminescence signal from cells treated with CHX was standardized against the signal from untreated cells to determine the residual ERAD substrates throughout the CHX chase. The rate of ERAD was computed by fitting a curve using an exponential function.

To assess the stability of mature ∆F508-CFTR-Nluc(CT), BEAS-2B Tet-on cells stably expressing ∆F508-CFTR-Nluc(CT) underwent transfection with siRNA (25 nM). Following transfection, the cells were treated with 1 µg/mL Dox and Trikafta (3 μM VX-661, 1 μM VX-445, 1 μM VX-770) at 37 °C for 2 days. The cellular stability of mature ∆F508-CFTR-Nluc(CT) was measured using previously established methods [23]. The administration of Trikafta was sustained throughout the CHX chase.

### 2.10. Pull-Down Experiments

In order to observe the interaction between HBH-∆F508-CFTR-3HA and FLAG-His-UBE3C, 293MSR cells that consistently expressed HBH-∆F508-CFTR-3HA were subjected to transfection with FLAG-His-UBE3C. Cells were treated with 2 mM sodium butyrate (NaB) for 1 day. Two days post-transfection, the cells were exposed to 10 µM MG-132 for 1 h and subsequently dissolved in a mild lysis buffer (150 mM NaCl, 20 mM Tris, 0.1% NP-40, pH 7.4) supplemented with 1 mM PMSF, 5 µg/mL leupeptin, and pepstatin. Next, the cell lysates were mixed with NeutrAvidin agarose (ThermoFisher) and left to incubate for 2 h at 4 °C. Following four washes with mild lysis buffer, the complex was separated using urea elution buffer (8 M urea, 2% SDS, 3 mM biotin) at 30 °C for 30 min and analyzed using Western blotting.

### 2.11. Protein Purification

His_6_-sumo-USP21 and GST-TUBE were expressed in the BL21 rosetta2 E. coli strain (EMD Millipore, Billerica, MA, USA). Cells were lysed by incubation with 1 mg/mL lysozyme for 30 min on ice, followed by sonication. The His-tagged proteins and GST-tagged proteins were purified using Ni-affinity and glutathione-affinity chromatography, respectively, as described [7,22].

### 2.12. Ub ELISA

CFTR ubiquitination levels in 293MSR cells were performed as previously [22,27]. 293MSR WT or RNF5/185 DKO cells stably expressing HBH-∆F508-CFTR underwent transfection with the specified siRNA. At 4 days post-transfection, cells were treated with 10 µM MG-132 (Cayman Chemical, Ann Arbor, MI, USA) for 3 h before cell lysis in RIPA buffer supplemented with supplemented with 5 µg/mL leupeptin, 5 µg/mL pepstatin A, 1 mM PMSF, 10 µM MG-132, and 5 mM N-Ethylmaleimide (NEM, FUJIFILM Wako Pure Chemical Corporation Cat# 054-02063). The HBH-∆F508-CFTR present in the cell lysate was fixed onto NeutrAvidin-coated 96-well white plates and then exposed to denaturation in 8 M urea at room temperature for 5 min. After 0.1% BSA blocking following 3 washes with 0.1% NP-40-PBS, the CFTR ubiquitination was detected by anti-K48 Ub (clone Apu2 ZooMAb, Sigma-Aldrich) antibody and quantified with HRP-conjugated secondary antibody. Alternatively, the ubiquitination was detected by using 10–50 µg/mL GST-TUBE and anti-GST antibody (clone 5A7, FUJIFILM Wako Pure Chemical Corporation). The CFTR ubiquitination levels were normalized for the CFTR level quantified by the anti-HA antibody (16B12, BioLegend, San Diego, CA, USA, Cat# 901515). For the USP21 digestion, the immobilized HBH-∆F508-CFTR on the plate was incubated with 5 µM His_6_-sumo-USP21 at 37 °C for 1 h before denaturation with 8 M urea. After the USP21 treatment, the plate was washed four times with 0.1% NP40-PBS, and then the immobilized CFTR was denatured in 8 M urea and used for the antibody reaction.

### 2.13. CFTR Ubiquitination Measurement by Western Blotting

CFTR ubiquitination levels in 293MSR cells were performed as previously [19,22]. 293MSR WT and RNF5/185 DKO cells transfected with siRNA (25 nM) were treated with 10 µM MG-132 for 3 h at 37 °C at 4 days post-transfection. Cells were then lysed in RIPA buffer supplemented with 5 µg/mL pepstatin, 5 µg/mL leupeptin, 1 mM PMSF, 10 µM MG-132, and 5 mM NEM, and HBH-∆F508-CFTR was purified using NeutrAvidin agarose (ThermoFisher) under denaturing conditions and analyzed by Western blotting with anti-Ub (P4D1) and anti-HA antibodies. The CFTR ubiquitination level was measured by densitometry and normalized for the CFTR level in the precipitate.

To detect the CFTR ubiquitination upon the UBE3C OE, 293MSR cells stably expressing HBH-∆F508-CFTR-3HA were transfected with Myc-Ub and FLAG-His-UBE3C. Cells were treated with 10 µM MG-132 for 3 h at 37 °C for 2 days post-transfection. Cells were then lysed in RIPA buffer supplemented with 5 µg/mL pepstatin, 5 µg/mL leupeptin, 1 mM PMSF, 10 µM MG-132, and 5 mM NEM, and HBH-∆F508-CFTR was purified using NeutrAvidin agarose under denaturing conditions and analyzed by Western blotting with anti-Myc and anti-HA antibodies.

### 2.14. Immunocytochemistry

293MSR cells stably expressing HBH-∆F508-CFTR grown on coverslips underwent transfection with FLAG-His-UBE3C. The following day, the cells were fixed using 4% paraformaldehyde for 20 min and then permeabilized with 0.1% Triton X-100 in PBS for 5 min. After blocking with 0.5% BSA in PBS for 30 min, the cells were incubated with anti-HA (16B12, BioLegend) and anti-UBE3C (Abcepta, Inc.) antibodies in 0.5% BSA in PBS for 1 h. Subsequently, cells were incubated with Alexa Fluor^®^ 488 AffiniPure Goat Anti-Mouse IgG (H+L) (Jackson Immuno Research) and Alexa Fluor^®^ 594 AffiniPure Goat Anti-Mouse IgG (H+L) (Jackson Immuno Research) antibodies in 0.5% BSA for an additional 1 h. To visualize the nuclei, cells were treated with Cellstain^®^ DAPI solution (Dojindo Laboratories, Kumamoto, Japan) for 5 min and then mounted using VECTASHIELD mounting medium (VECTOR Laboratories, Newark, CA, USA). Single optical sections were captured using an inverted laser confocal fluorescence microscope (SP8, Leica, Tokyo, Japan) equipped with an HC PL APO 63×/NA 1.40 objective.

### 2.15. Statistical Analysis

For quantification, data from at least three independent experiments were used, where the data are expressed as means ± standard error (SE). Statistical significance was assessed by either a two-tailed unpaired Student’s *t*-test, a one-way analysis of variance (ANOVA) with Dunnett’s multiple comparison test, or a two-way ANOVA with Holm-Sidak multiple comparison tests performed using GraphPad Prism 8. A *p* value < 0.05 was defined as statistically significant.

## 3. Results

### 3.1. UBE3C Limits Cell Surface Expression of ∆F508-CFTR

**Figure 1:**
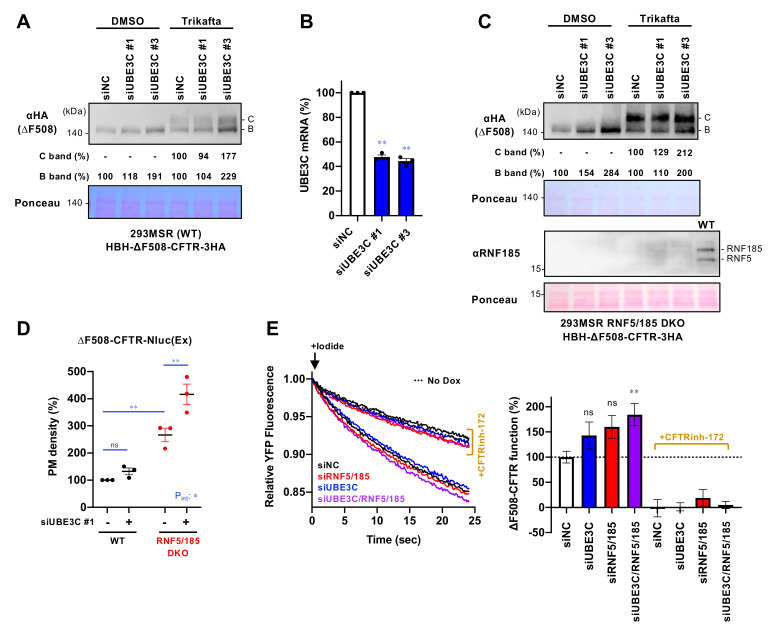
UBE3C limits the abundance of ∆F508-CFTR. (**A**) Western blotting shows HBH-∆F508-CFTR-3HA level in 293MSR cells transfected with 25 nM siNC, siUBE3C #1, or siUBE3C #3. Cells were treated with or without Trikafta (3 µM VX-661, 1 µM VX-445, 1 µM VX-770) for 1 day. The quantities of immature (B band) and mature (C band) ΔF508-CFTR were quantified using densitometry and presented as a percentage relative to the control. Cell lysates were prepared 4 days post-transfection. Ponceau staining was used as a loading control. (**B**) RT-qPCR measured UBE3C KD in 293MSR cells at 2 days post-transfection of siRNA (25 nM). Statistical significance was evaluated using a one-way repeated-measures (RM) ANOVA alongside Dunnett’s multiple comparison tests (n = 3). (**C**) Western blotting shows HBH-∆F508-CFTR-3HA level in RNF5/185 DKO cells as performed as A. Ablation of RNF5 and RNF185 was confirmed by an anti-RNF185 antibody that recognizes both RNF5 and RNF185 and the lysate from 293MSR cells (WT) was employed to confirm the presence of bands corresponding to RNF185 and RNF5. (**D**) PM level of ∆F508-CFTR-Nluc(Ex) in 293MSR WT and RNF5/185 DKO cells with 25 nM siNC (-) or siUBE3C #1 (+) (n = 3). Cells were treated with Trikafta (3 µM VX-661, 1 µM VX-445, 1 µM VX-770) for 1 day. A two-way RM ANOVA with Holm-Sidak multiple comparison tests demonstrated a significant interaction between UBE3C KD and RNF5/185 DKO (Pint < 0.05). (**E**) The channel function of ∆F508-CFTR-3HA in CFBE Teton cells transfected with siRNA indicated and treated with Trikafta (3 µM VX-661, 1 µM VX-445, 1 µM VX-770) for 2 days was evaluated using a YFP quenching assay. The rate of initial YFP quenching was measured and quantified as the CFTR function (right, n = 10–12). The total siRNA concentration was adjusted to 125 nM (25 nM siUBE3C #1, 50 nM siRNF5 pool, and 50 nM siRNF185 pool) for all samples. The assay was conducted 4 days post-transfection. Statistical significance was assessed by a one-way ANOVA with Dunnett’s multiple comparison tests. Data represent mean ± SE. * *p* < 0.05, ** *p* < 0.01, ns, not significant.

To investigate UBE3C’s involvement in CFTR protein QC, we assessed the impact of UBE3C KD on the protein level of ∆F508-CFTR in 293MSR cells that stably expressed HBH-∆F508-CFTR-3HA. This modified CFTR protein is fused with an N-terminal histidine-biotin-histidine (HBH) tag, and it also has a 3xHA tag in the 4th extracellular loop [22]. In our Western blot analysis, we observed that UBE3C KD, using siUBE3C #1 or siUBE3C #3, led to a slight increase in immature ∆F508-CFTR (Figure 1A). We confirmed the reduction of endogenous UBE3C under these conditions through quantitative RT-PCR (Figure 1B). To explore whether the increased levels of ∆F508-CFTR due to UBE3C KD could mature into the fully functional form in post-ER compartments, we treated the cells with the CF drug Trikafta, which consists of CFTR correctors VX-661 and VX-445, as well as the CFTR potentiator VX-770 [28]. Western blot analysis revealed that Trikafta treatment led to the appearance of mature ∆F508-CFTR, and this effect was slightly more pronounced when UBE3C was knocked down, especially when using siUBE3C #3 (Figure 1A). These findings suggest that UBE3C KD may increase the pool of ∆F508-CFTR in the ER, which can then mature into the functional form at the Golgi apparatus.

In a previous study, it was suggested that UBE3C operates downstream of the RNF185/MBRL complex [15]. To investigate whether UBE3C regulates ∆F508-CFTR levels through the RNF185-mediated ERAD pathway, we used 293MSR cells where both RNF185 and its functional paralog, RNF5, were ablated [24]. As previously demonstrated, we observed a significant reduction in the ERAD of ∆F508-CFTR in RNF5/185 double knockout (DKO) cells [24]. Our Western blot analysis indicated that UBE3C KD using siUBE3C #1 or siUBE3C #3 increased the levels of immature ∆F508-CFTR, even in the RNF5/185 DKO cells (Figure 1C). After treatment with Trikafta, UBE3C KD also led to an increase in the mature form of ∆F508-CFTR in RNF5/185 DKO cells (Figure 1C). These results suggest that UBE3C may regulate the abundance of ∆F508-CFTR through a mechanism independent of RNF5/185.

Subsequently, we determined whether UBE3C KD increased the cell surface expression of ∆F508-CFTR. We quantified the cell surface level of ∆F508-CFTR-Nanoluc (Nluc)(Ex), which had the Nluc tag inserted into the 4th extracellular loop of CFTR [23]. To stimulate the PM expression of ∆F508-CFTR, we treated the cells with the CF drug Trikafta. The cell surface level of ∆F508-CFTR-Nluc(Ex) was measured using a cell-impermeable Nluc substrate. In our Nluc assay, we observed that UBE3C KD led to a marginal increase in the cell surface ∆F508-CFTR-Nluc(Ex) in 293MSR WT cells (Figure 1D). However, in the RNF5/185 DKO cells, UBE3C KD significantly enhanced the cell surface expression of ∆F508-CFTR compared to the 293MSR WT cells (Figure 1D). To assess whether the increased cell surface ∆F508-CFTR was functionally acting as a regulated Cl^-^ channel, we conducted a halide-sensitive YFP quenching assay in CFBE Tet-on cells that stably expressed YFP-H148Q/I152L/F46 and inducible ∆F508-CFTR-3HA [22]. As expected, UBE3C KD and RNF5/185 DKD had an additive effect on the channel function of ∆F508-CFTR-3HA (Figure 1E). This enhanced YFP quenching was inhibited by a CFTR inhibitor-172 (CFTRinh-172), confirming that the YFP quenching resulted from CFTR channel activity (Figure 1E). In summary, these findings indicate that UBE3C restricts the presence of ∆F508-CFTR as a functional Cl^-^ channel on the PM, even in the absence of RNF5 and RNF185, especially when triggered by Trikafta treatment.

### 3.2. UBE3C Facilitates ERAD of ∆F508-CFTR

**Figure 2:**
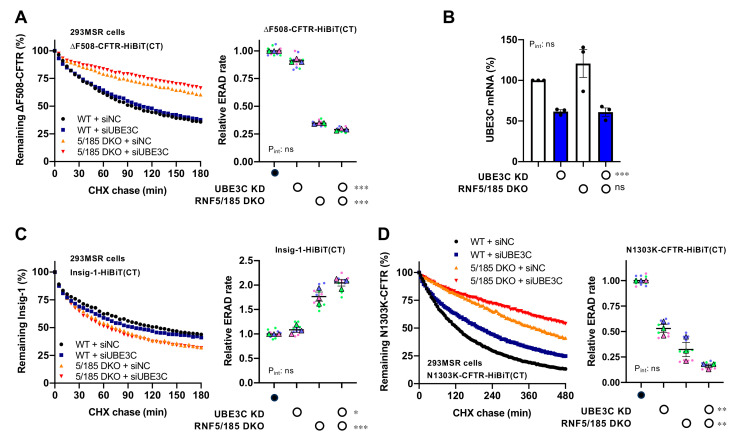
UBE3C attenuates ∆F508-CFTR ERAD. (**A**) The degradation kinetics of ∆F508-CFTR-HiBiT(CT) in 293MSR WT and RNF5/185 KO cells transfected with 25 nM siNC or siUBE3C #1. Luminescence levels were consistently observed for 180 min while cells were exposed to 100 µg/mL CHX. The data were plotted after normalization to the luminescence levels of untreated cells. The ERAD rate of ∆F508-CFTR-HiBiT(CT) was determined by fitting individual kinetic degradation curves (right, n = 3). Two-way ANOVA demonstrated a notable primary impact of UBE3C KD or RNF5/185 DKO. However, it did not reveal any interaction between the two factors (P_int_). (**B**) UBE3C KD in 293MSR WT and RNF5/185 cells was validated using RT-qPCR (n = 3). Two-way ANOVA demonstrated a significant main effect of UBE3C KD but not of RNF5/185 DKO, with no observed interaction between the two factors (P_int_). (**C**,**D**) Kinetic degradation of Insig-1-HiBiT(CT) ((**C**), n = 3) and N1303K-CFTR-HiBiT(CT) ((**D**), n = 3) in 293MSR WT and RNF5/185 KO cells transfected with 25 nM siNC or siUBE3C #1. The ERAD rate was calculated by fitting each kinetic degradation curve (right). Two-way ANOVA demonstrated a significant main effect of UBE3C KD or RNF5/185 DKO but no interaction between the two factors (P_int_). Each biological replicate (n) is color-coded: the averages from 4 technical replicates are shown in triangles (**A**,**C**,**D**). Statistical significance was assessed by a two-way RM ANOVA (**A**,**C**,**D**) or two-way ANOVA (**B**). Data represent mean ± SE. * *p* < 0.05, ** *p* < 0.01, *** *p* < 0.001, ns, not significant.

To understand how UBE3C restricts ∆F508-CFTR levels, we examined the impact of UBE3C KD on ERAD. We recently developed a HiBiT-based ERAD assay, allowing precise measurement of the degradation kinetics of various ERAD substrates [24]. Using this novel assay, we assessed the effect of UBE3C KD on the ERAD of ∆F508-CFTR-HiBiT(CT) in 293MSR cells. As anticipated, UBE3C KD modestly slowed down ERAD, but it additively reduced the ERAD rate of ∆F508-CFTR in combination with RNF5/185 DKO (Figure 2A). We verified that siUBE3C#1 effectively decreased UBE3C mRNA in both 293MSR WT and RNF5/185 DKO cells (Figure 2B). In contrast, UBE3C KD did not affect the ERAD of Insig-1-HiBiT(CT) in either WT or RNF5/185 DKO cells (Figure 2C). Rather, UBE3C KD, along with RNF5/185 DKO, enhanced the ERAD of Insig-1, which is a similar phenotype observed previously [25] (Figure 2C). We also explored the influence of UBE3C KD on the ERAD of N1303K-CFTR, which is recognized for its NBD2 mutation causing the unfolding of MSD1 and MSD2 [29]. As expected, UBE3C KD, combined with RNF5/185 DKO, additively reduced the ERAD of N1303K-CFTR-HiBiT(CT) (Figure 2D). These results suggest that UBE3C limits the levels of misfolded CFTR by promoting ERAD, and this effect is maintained even in the absence of RNF5/185.

### 3.3. UBE3C Reduces ∆F508-CFTR Independently of Its E3 Ligase Activity

**Figure 3:**
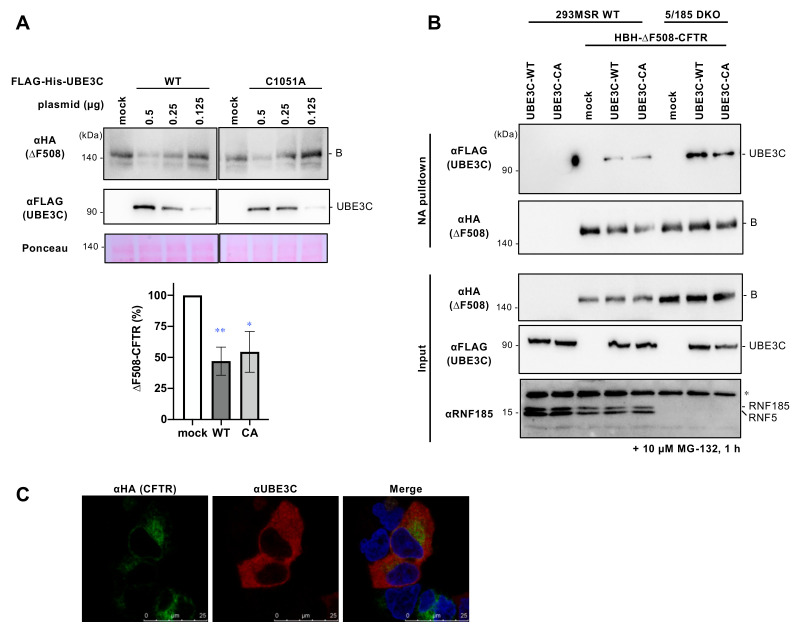
UBE3C OE reduces ∆F508-CFTR abundance independently of its E3 ligase activity. (**A**) Western blotting shows ∆F508-CFTR-3HA level in 293MSR cells co-transfected with ∆F508-CFTR-3HA and FLAG-His-UBE3C WT or C1051A (0.5, 0.25, or 0.125 µg). Cells were treated with 2 mM NaB for 1 day before cell lysis. The immature ΔF508-CFTR (B band) in cells transfected with 0.5 µg FLAG-His-UBE3C WT or C1051A (CA) was quantified using densitometry (n = 3–4). Ponceau staining was used as a loading control. Statistical significance was assessed by one-way ANOVA. Data represent mean ± SE. * *p* < 0.05, ** *p* < 0.01. (**B**) The interaction of FLAG-His-UBE3C WT or C1051A mutant with HBH-ΔF508-CFTR-3HA in 293MSR WT and RNF5/185 DKO cells was analyzed by NA pull-down and Western blotting. Cells were treated with 2 mM NaB for 1 day and 10 µM MG-132 for 1 h before cell lysis. The asterisk shows a non-specific band. (**C**) Cellular localization of HBH-∆F508-CFTR-3HA and FLAG-His-UBE3C in 293MSR cells transfected with FLAG-His-UBE3C. Cells were treated with 10 µM MG-132 for 1 h before fixation. The nucleus was stained with DAPI.

To investigate whether UBE3C regulates ∆F508-CFTR through its E3 ligase activity, we generated a catalytically inactive mutant, C1051A-UBE3C [30]. In Western blot analysis, we observed that overexpression (OE) of FLAG-His-UBE3C reduced the abundance of ∆F508-CFTR-3HA in a dose-dependent manner (Figure 3A). Surprisingly, the OE of FLAG-His-C1051A-UBE3C also reduced ∆F508-CFTR levels (Figure 3A). This finding implies that UBE3C’s E3 ligase activity is not required for reducing ∆F508-CFTR levels.

Next, we explored the physical interaction between UBE3C and ∆F508-CFTR in 293MSR cells expressing HBH-∆F508-CFTR-3HA and FLAG-His-UBE3C. NeutrAvidin (NA) pull-down experiments revealed that FLAG-His-UBE3C and its catalytically inactive C1051A mutant interacted with HBH-∆F508-CFTR-3HA in 293MSR (Figure 3B). Similar results were observed in the RNF5/185 DKO cells (Figure 3B). Thus, UBE3C appears to interact with ∆F508-CFTR at the ER, independent of RNF5/185.

We further performed immunocytochemistry to examine the cellular localization of UBE3C. We treated cells with a proteasome inhibitor, MG-132, to prevent the degradation of ∆F508-CFTR upon UBE3C OE. The transfected UBE3C was observed to be widely present in the cytoplasm and was partially colocalized with ∆F508-CFTR at the perinuclear region, supporting their interaction at the ER (Figure 3C).

### 3.4. UBE3C Has a Limited Impact on the Ubiquitination of ∆F508-CFTR

**Figure 4:**
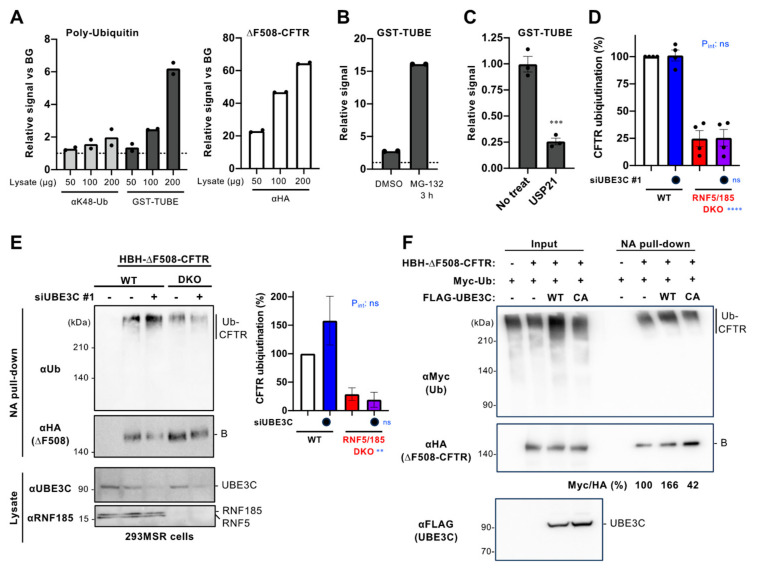
The role of UBE3C in the ∆F508-CFTR ubiquitination. (**A**) Polyubiquitination of HBH-∆F508-CFTR-3HA in BHK cells was detected by ELISA using an anti-K48-linked polyubiquitin antibody (αK48-Ub, Apu2 ZooMAb) or GST-TUBE and an anti-GST antibody (n = 2). The CFTR amount on the ELISA plate was also quantified using an anti-HA antibody (right). The signal from the sample containing HBH-∆F508-CFTR-3HA was calculated relative to the BG signal obtained from the sample derived from CFTR-non-expressing cells. The cells underwent treatment with 10 µM MG-132 for a duration of 3 h before undergoing cell lysis. (**B**) The GST-TUBE binding signal exhibited an increase in the case of HBH-∆F508-CFTR-3HA from BHK cells treated with 10 µM MG-132 for 3 h (n = 2). Cell lysates (150 µg) were used for analysis. (**C**) The signal detected by GST-TUBE was reduced by in vitro digestion by 5 µM USP21 for 1 h at 37°C prior to the GST-TUBE binding (n = 3). Cell lysates (150 µg) were used for analysis. (**D**) The ubiquitination levels of HBH-∆F508-CFTR-3HA in 293MSR WT and RNF5/185 DKO cells transfected with 25 nM siRNA indicated were measured by ELISA using GST-TUBE (n = 4). The cells underwent treatment with 10 µM MG-132 for a duration of 3 h before undergoing cell lysis. A two-way ANOVA demonstrated a significant main effect of RNF5/185 DKO but not of UBE3C KD, with no observed interaction between the two factors (P_int_). (**E**) The ubiquitination level of HBH-∆F508-CFTR-3HA in 293MSR WT and RNF5/185 DKO cells transfected with 25 nM siNC (-) or siUBE3C #1 (+) was measured by NA pull-down and normalized for CFTR in precipitates. The cells underwent treatment with 10 µM MG-132 for a duration of 3 h before undergoing cell lysis. The levels of CFTR ubiquitination were assessed using densitometry and presented as a percentage relative to the control group (right, n = 3). Two-way ANOVA demonstrated a significant main effect of RNF5/185 DKO but not of UBE3C KD, with no observed interaction between the two factors (P_int_). (**F**) Ubiquitination level of HBH-∆F508-CFTR-3HA in 293MSR cells transfected with Myc-Ub and FLAG-His-UBE3C WT or C1051A mutant was measured as E. Cells were treated with 2 mM NaB for 1 day and 10 µM MG-132 for 3 h before cell lysis. The levels of CFTR ubiquitination (Myc/HA) were quantified using densitometry and reported as a percentage in comparison to the control. Data represent the mean (**A**,**B**) or mean ± SE (**C**,**D**,**E**). Statistical significance was assessed by an unpaired *t*-test (**C**) or two-way ANOVA (**D**,**E**). ** *p* < 0.01, *** *p* < 0.001, **** *p* < 0.0001, ns, not significant.

To determine the role of UBE3C in CFTR ubiquitination, we conducted a Ub ELISA using an anti-K48-linked polyUb chains (K48-Ub) antibody [22,27]. Unlike the K48-Ub (Apu2) antibody, which is no longer commercially available, we detected a faint signal of ∆F508-CFTR ubiquitination using the new K48-Ub (Apu2 ZooMAb) antibody (Figure 4A). Additionally, we utilized GST-TUBE (Tandem Ub Binding Entity) [31,32] to detect CFTR ubiquitination. The GST-TUBE, derived from the UBA domain of UBQLN1, which serves as a proteasome-shuttling factor, has been reported not to exhibit any preference for poly-Ub chain linkage [33,34]. We successfully observed the specific binding of GST-TUBE to the immobilized ∆F508-CFTR on the NeutrAvidin plate and obtained an approximately three-fold stronger signal compared to that of the K48-Ub (Apu2 ZooMAb) antibody (Figure 4A). The signal obtained with GST-TUBE significantly increased in the ∆F508-CFTR treated with the proteasome inhibitor MG-132, which leads to the accumulation of CFTR polyubiquitination [22] (Figure 4B). Furthermore, pretreatment with recombinant USP21, a non-selective deubiquitinase (DUB) [35,36], substantially reduced the binding of GST-TUBE to immobilized HBH-∆F508-CFTR (Figure 4C). These results indicate that the binding of GST-TUBE to ∆F508-CFTR depends on ubiquitination, and, therefore, TUBE binding can be used to quantify the level of CFTR ubiquitination. We employed the TUBE-ELISA we established to assess the influence of UBE3C KD on the ubiquitination of HBH-∆F508-CFTR in 293MSR cells. In line with our recent study [24], the RNF5/185 DKO led to a substantial decrease in TUBE binding, indicating reduced ∆F508-CFTR ubiquitination at the ER (Figure 4D). However, in contrast, UBE3C KD did not seem to have an impact on TUBE binding in both WT and RNF5/185 DKO cells (Figure 4D).

To further validate the involvement of UBE3C in CFTR ubiquitination, we conducted a traditional Western blotting analysis on the isolated HBH-∆F508-CFTR-3HA from 293MSR cells. Western blotting showed that consistent with the findings from the TUBE ELISA, UBE3C KD failed to reduce the ubiquitination of ∆F508-CFTR in both WT and RNF5/185 DKO cells (Figure 4E). We observed reduced CFTR ubiquitination in the RNF5/185 DKO cells compared to the WT cells, which aligns with the TUBE-ELISA results (Figure 4E). These findings suggest that UBE3C may be involved in the ERAD of misfolded CFTR through mechanisms other than CFTR ubiquitination. We also investigated the impact of overexpressing UBE3C on the ubiquitination of HBH-∆F508-CFTR in 293MSR cells co-expressing Myc-Ub. Our analysis revealed a slight increase in the ubiquitination of ∆F508-CFTR upon OE of FLAG-His-UBE3C (Figure 4F). In contrast, the OE of the catalytically inactive C1051A-UBE3C mutant did not increase ubiquitination (Figure 4F). These results imply that UBE3C may possess the ability to increase the ubiquitination of misfolded CFTR at the ER.

### 3.5. UBE3C Facilitates ERAD of Misfolded ABCB1

**Figure 5:**
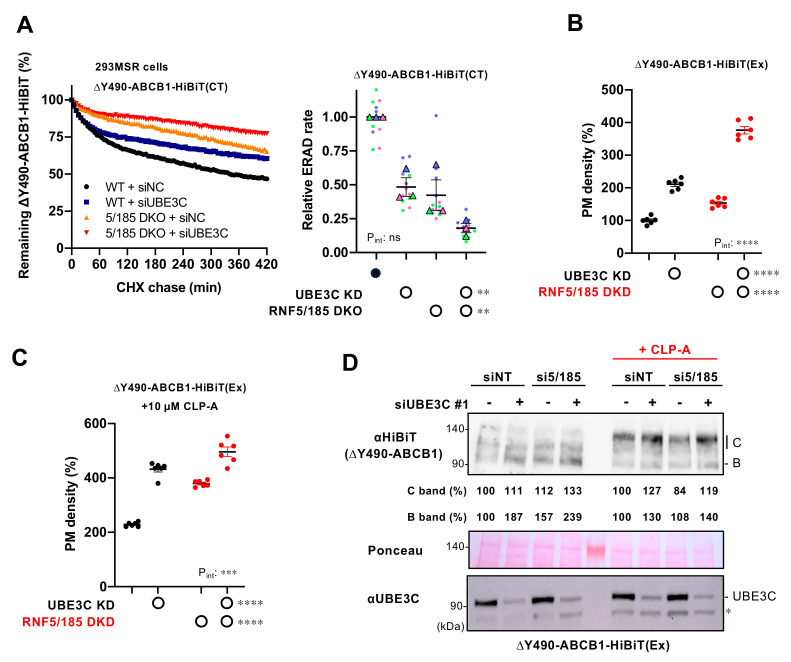
UBE3C facilitates the ERAD of misfolded ABCB1. (**A**) The HiBiT degradation assay quantified the ERAD of ΔY490-ABCB1-HiBiT(CT) in 293MSR WT and RNF5/185 DKO cells after transfection with either 25 nM siNC or siUBE3C #1. Luminescence was continually tracked for 420 min while CHX was present, and the data were graphed after normalization to the luminescence levels of non-treated cells. The ERAD rate of ΔY490-ABCB1-HiBiT(CT) was determined by fitting individual kinetic degradation curves (right, n = 3). Each biological replicate (n) is color-coded; the averages from 4 technical replicates are shown in triangles. Two-way ANOVA demonstrated a significant effect of UBE3C KD or RNF5/185 DKO but no interaction between the two factors (P_int_). (**B**,**C**) PM levels of ∆Y490-ABCB1-HiBiT(Ex) in BEAS-2B cells transfected with 25 nM siUBE3C #1 and/or 50 nM siRNF5 and 50 nM siRNF185 (RNF5/185 DKD) as indicated (n = 6). (**C**) Cells were treated with 10 µM CLP-A for 24 h to facilitate cell surface expression. Two-way ANOVA demonstrated a significant effect of UBE3C KD or RNF5/185 DKO, as well as an interaction between the two factors (P_int_). (**D**) Western blotting shows ∆Y490-ABCB1-HiBiT(Ex) level in BEAS-2B cells transfected with 25 nM siNC (-) or siUBE3C #1 (+), 100 nM siNT, or 50 nM siRNF5 and siRNF185 (si5/185). Cells were subjected to a 24-h treatment with or without 10 µM CLP-A. Additionally, cells were treated with 2 mM NaB for 24 h before cell lysis. The levels of immature (B band) and mature (C band) ∆Y490-ABCB1 were quantified using densitometry and presented as a percentage relative to the control. Cell lysates were prepared at 4 days post-transfection. Ponceau staining was employed as a loading control. The asterisk shows a non-specific band. Statistical significance was evaluated through a two-way RM ANOVA (**A**) or two-way ANOVA (**B**,**C**). Data represent mean ± SE. * *p* < 0.05, ** *p* < 0.01, *** *p* < 0.001, **** *p* < 0.0001, ns, not significant.

Given that ∆F508-CFTR is considered an ERAD-C substrate that has lesions in the cytoplasm [37,38], UBE3C may facilitate the degradation of various ERAD-C substrates, as previously reported [39]. To investigate this possibility, we employed ∆Y490-ABCB1, which carries a deletion of Y490 in the cytosolic NBD1 and shares a similar mutation pattern to the ∆F508 mutation in CFTR [40]. In our HiBiT degradation assay, we found that UBE3C KD significantly slowed down the ERAD of ∆Y490-ABCB1-HiBiT(CT) in 293MSR WT cells, similar to the effect observed in RNF5/185 DKO cells [24] (Figure 5A). Furthermore, UBE3C KD, in combination with RNF5/185 DKO, led to an even further reduction in the ERAD rate of ∆Y490-ABCB1-HiBiT(CT) (Figure 5A). Consequently, ablation of UBE3C or RNF5/185 increased the cell surface expression of ∆Y490-ABCB1-HiBiT(Ex), where the HiBiT tag was inserted in the 1st extracellular loop of ABCB1 (Figure 5B). Additionally, UBE3C KD enhanced the cell surface ∆Y490-ABCB1-HiBiT(Ex) even more in RNF5/185 DKD cells (Figure 5B). These effects were also observed in the presence of cyclosporin A (CLP-A), which is a ligand of ABCB1 and promotes the cell surface expression of ∆Y490-ABCB1 [41] (Figure 5C). Western blotting further validated these findings, as UBE3C KD or RNF5/185 DKD appeared to increase the ∆Y490-ABCB1-HiBiT(Ex), and their combined effect was even more pronounced, especially in the absence of CLP-A (Figure 5D). Additionally, we observed that UBE3C KD resulted in an increase in the mature form of ∆Y490-ABCB1-HiBiT(Ex) upon CLP-A treatment in both siNT- and siRNF5/185-transfected cells (Figure 5D). These findings suggest that UBE3C might be involved in the ERAD of ∆Y490-ABCB1, even within the RNF5/185-independent ERAD pathway.

### 3.6. UBE3C Participates in Peripheral CFTR Quality Control

**Figure 6:**
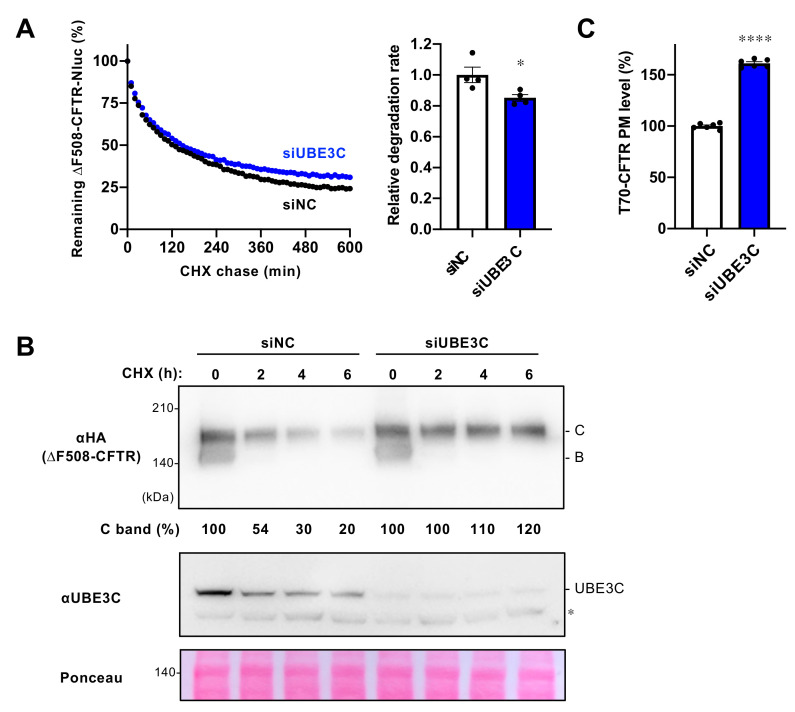
UBE3C participates in peripheral CFTR quality control. (**A**) Representative traces of mature ΔF508-CFTR-Nluc(CT) elimination in BEAS-2B cells transfected with 25 nM siNC or siUBE3C #1. Cells were pre-treated with 1 µg/mL Dox and Trikafta (3 μM VX-661, 1 μM VX-445, 1 μM VX-770) at 37 °C for 2 days. During the CHX chase, Trikafta was also treated. The degradation rate of ΔF508-CFTR-Nluc(CT) was determined by fitting individual kinetic degradation curves (right, n = 4). (**B**) Western blotting with CHX chase measured the stability of mature ∆F508-CFTR-3HA in 293MSR cells transfected with 25 nM siNC or siUBE3C #3. Cells were pre-treated with Trikafta (3 μM VX-661, 1 μM VX-445, 1 μM VX-770) and 2 mM NaB at 37 °C for 24 h but did not during the 100 µg/mL CHX chase. Levels of immature (B band) and mature (C band) ∆F508-CFTR were quantified using densitometry and reported as a percentage relative to the control. Cell lysates were prepared 4 days post-transfection. Ponceau staining was employed as a loading control. The asterisk shows a non-specific band. (**C**) PM levels of T70-CFTR-HiBiT(Ex) in BEAS-2B cells transfected with 25 nM siNC or siUBE3C #1 (n = 6). Statistical significance was assessed by an unpaired *t*-test (**A**,**C**). Data represent mean ± SE. * *p* < 0.05, **** *p* < 0.0001.

Given that UBE3C is localized in the cytoplasm [42], it is plausible that UBE3C may interact with misfolded CFTR not only at the ER but also at post-Golgi compartments, including the PM. Furthermore, the elevated PM levels of ∆F508-CFTR observed upon UBE3C KD in the presence of Trikafta, which does not fully stabilize ∆F508-CFTR on the PM due to a substantial portion of the mutant CFTR still being subject to ubiquitination and degradation [23,43], may be indicative of its role in peripheral CFTR QC [19,22]. To investigate whether UBE3C is associated with CFTR peripheral QC, we assessed the impact of UBE3C KD on the stability of mature ∆F508-CFTR-Nluc [23]. We treated BEAS-2B cells stably expressing ∆F508-CFTR-Nluc with Trikafta to stimulate the maturation of ∆F508-CFTR. Our continuous CFTR-Nluc luminescence measurements revealed that the half-life of ∆F508-CFTR after Trikafta treatment was significantly longer, approximately 2 h (Figure 6A), compared to the immature ∆F508-CFTR located in the ER, which had a half-life of approximately 0.5 h [23]. This suggests that the assay is specifically measuring the degradation of mature ∆F508-CFTR located in post-Golgi compartments, as reported previously [23]. Similar to RFFL KD [23], UBE3C KD led to a slight but significant reduction in the degradation of mature ∆F508-CFTR-Nluc (Figure 6A). In line with this observation, Western blot analysis using a CHX chase experiment demonstrated that UBE3C KD resulted in increased stability of the mature form of ∆F508-CFTR-3HA induced by Trikafta treatment (Figure 6B). This indicates that UBE3C plays a role in promoting the peripheral degradation of mature ∆F508-CFTR. Furthermore, we examined the effect of UBE3C KD on T70-CFTR, a class VI CFTR mutant known for accelerated PM turnover [18,44]. As expected, UBE3C KD elevated the PM levels of T70-CFTR-HiBiT(Ex) in BEAS-2B cells (Figure 6C). These results strongly suggest that cytoplasmic UBE3C contributes to the removal of conformationally defective CFTR, even at post-Golgi compartments, and participates in CFTR peripheral QC.

## 4. Discussion

In this study, we have unveiled the role of the cytosolic E3 ligase UBE3C in the ERAD process, specifically in the degradation of misfolded CFTR and ABCB1. Furthermore, UBE3C’s involvement extends to the RNF5/185-independent ERAD pathway. Notably, the combined effect of UBE3C KD and RNF5/185 DKO/DKD results in a synergistic increase in the levels of ∆F508-CFTR and ∆Y490-ABCB1 on the PM, further supporting UBE3C’s role in the RNF5/185-independent ERAD process. However, in the presence of CLP-A, the enhanced effect of UBE3C KD was slightly diminished in RNF5/185 DKD cells, suggesting that the conformation of the ABCB1 mutant rescued by CLP-A and RNF5/185 DKD may render it less susceptible to protein QC by UBE3C.

The function of UBE3C in the ERAD of misfolded membrane proteins shares similarities with another E3 ligase, HERC3 [24]. Both UBE3C and HERC3 are HECT-type E3 ligases primarily localized in the cytoplasm [45], and both contribute to the ERAD of ∆F508-CFTR in the RNF5/185-independent ERAD pathway [24]. In contrast to HERC3, UBE3C has a broader range of action, as it is also involved in the ERAD of ∆Y490-ABCB1. Although the specific mechanisms by which UBE3C and HERC3 recognize their substrates are not yet clear, UBE3C seems to have a unique function distinct from HERC3. While HERC3 is thought to sense the MSDs [24], UBE3C might detect conformational defects in the cytoplasmic region of ER membrane proteins. This hypothesis aligns with previous research suggesting that UBE3C is essential for the efficient degradation of certain ERAD-C substrates [40]. Considering the observed conformational defects in the CFTR MSDs in the case of ∆F508-CFTR [6,46,47] and the unfolding of MSDs triggered by the NBD2 mutant N1303K-CFTR [29], we cannot exclude the possibility that UBE3C may also possess the capability to identify conformational issues in the CFTR MSDs. Further research and investigations may be needed to validate this possibility.

Based on our experimental findings, UBE3C’s role in ∆F508-CFTR ERAD appears to be partial compared to RNF5/185, showing a similarity in contribution to the cytoplasmic E3 ligase HERC3 [24]. However, given UBE3C’s substantial impact on ∆Y490-ABCB1 ERAD, it suggests that UBE3C’s involvement varies based on the misfolding region or characteristics of the ERAD substrate. In forthcoming investigations, it is imperative to elucidate UBE3C’s substrate recognition mechanism to discern the structural abnormalities it identifies. This exploration may offer valuable insights into UBE3C’s role in ERAD.

Our OE experiments suggest that UBE3C has the potential to increase the ubiquitination of immature ∆F508-CFTR, depending on its catalytic activity. UBE3C is known to be involved in the formation of K29- and K48-linked polyubiquitin chains [48,49] and acts as an E4 enzyme [17]. This implies that UBE3C might modify and/or elongate the polyUb chains on misfolded CFTR. However, it is improbable that UBE3C promotes ∆F508-CFTR degradation through a mechanism dependent on ubiquitination, as its catalytically inactive mutant was able to reduce ∆F508-CFTR levels similarly to its wild-type counterpart. Moreover, our KD experiments indicate that UBE3C had a minimal impact on the ubiquitination of immature ∆F508-CFTR. The mechanism through which UBE3C promotes ERAD without impacting CFTR ubiquitination differs significantly from that of HERC3, a HECT-type E3 ligase involved in ERAD independent of RNF5/185 [24]. It has been demonstrated that UBE3C is recruited to the proteasome in response to the unfolding of cytosolic proteins [50] and serves as a positive regulator of proteasomal processivity by facilitating the degradation of partially degraded protein fragments that tend to accumulate [31]. Therefore, UBE3C may be involved in efficiently transporting misfolded CFTR and ABCB1 to the proteasome for degradation, even without relying on its E3 ligase activity. This mechanism aligns with a model in which certain Ub ligases directly present the substrate protein to the 20S proteasome, facilitating their degradation and essentially acting as ‘recruiters’ [51,52,53].

In this study, we have established the TUBE ELISA as a method for measuring CFTR ubiquitination. In our previous work, we developed a Ub ELISA using the K48-Ub (Apu2) antibody [22,27]. However, as the K48-Ub (Apu2) antibody became unavailable commercially, we tested a new K48-Ub (Apu2 ZooMAb) antibody in this study. Unfortunately, the new K48-Ub antibody did not provide satisfactory results for our Ub ELISA and only produced a weak signal. Consequently, we opted to use TR-TUBE, derived from the UBA domain of UBQLN1, as a probe for polyubiquitination [31,32]. Given the characteristics of UBQLN1’s UBA domain [34], GST-TUBE is likely to bind all types of Ub chains, including K63- and K48-linked chains, as observed in previous studies [32,54]. The TUBE ELISA we introduced in this study may prove valuable for detecting specific types of polyUb chains using the K63 chain-specific TUBE [55], as well as for investigating proteins that interact with the K29 chains [56].

Our results suggest the possibility that UBE3C is involved in the peripheral QC of CFTR. Several cytosolic E3 ligases, including CHIP [19], RFFL [22], and RNF34 [23] have been reported to participate in peripheral QC. Given that UBE3C directly interacts with its substrate and facilitates its substrate ubiquitination in vitro [42], it may directly interact with the cytosolic regions of misfolded CFTR at post-Golgi compartments. While more investigations are necessary, UBE3C may function as part of the chaperone-independent peripheral QC mechanism [22]. A previous study demonstrated that proteasome inhibitors reduced the removal of cell surface ∆F508-CFTR [57]. While the exact function of the proteasome in peripheral QC is not fully understood, it is possible that UBE3C plays a role in promoting proteasomal involvement in the degradation of misfolded CFTR in the post-Golgi compartments, including the PM. Although further investigation is needed to explore the potential adverse effects resulting from UBE3C inhibition, it’s evident that such inhibition suppresses ERAD and peripheral degradation of ∆F508-CFTR, consequently leading to an increase in functional CFTR channels. This observation suggests that targeting UBE3C could be a viable strategy in CF treatment, addressing both ER and peripheral CFTR QC mechanisms. This study reveals a new role for the UBE3C E3 ligase in the QC of membrane proteins, providing insights into previously unexplored aspects of UBE3C’s mechanism of action.

## Figures and Tables

**Table 1:** Primers used for quantitative RT-PCR.

Primer	Sequence
Human UBE3C-Forward	5′-TGGCCCCAACCTTACCCTT-3′
Human UBE3C-Reverse	5′-GCAGCAACCTGCAACAGAG-3′
Human GAPDH-Forward	5′-CATGAGAAGTATGACAACAGCCT-3′
Human GAPDH-Reverse	5′-AGTCCTTCCACGATACCAAAGT-3′

## Data Availability

The data presented in this study are available upon request from the corresponding author.

## References

[1] Riordan J.R. (2008). CFTR function and prospects for therapy. Annu. Rev. Biochem..

[2] Boucher R.C. (2004). New concepts of the pathogenesis of cystic fibrosis lung disease. Eur. Respir. J..

[3] Riordan J.R., Rommens J.M., Kerem B., Alon N., Rozmahel R., Grzelczak Z., Zielenski J., Lok S., Plavsic N., Chou J.-L. (1989). Identification of the cystic fibrosis gene: Cloning and characterization of complementary DNA. Science.

[4] Rich D.P., Anderson M.P., Gregory R.J., Cheng S.H., Paul S., Jefferson D.M., McCann J.D., Klinger K.W., Smith A.E., Welsh M.J. (1990). Expression of Cystic-Fibrosis Transmembrane Conductance Regulator Corrects Defective Chloride Channel Regulation in Cystic-Fibrosis Airway Epithelial-Cells. Nature.

[5] White M.B., Amos J., Hsu J.M.C., Gerrard B., Finn P., Dean M. (1990). A Frame-Shift Mutation in The Cystic-Fibrosis Gene. Nature.

[6] Okiyoneda T., Lukacs G.L. (2012). Fixing cystic fibrosis by correcting CFTR domain assembly. J. Cell Biol..

[7] Rabeh W.M., Bossard F., Xu H., Okiyoneda T., Bagdany M., Mulvihill C.M., Du K., di Bernardo S., Liu Y., Konermann L. (2012). Correction of Both NBD1 Energetics and Domain Interface Is Required to Restore Delta F508 CFTR Folding and Function. Cell.

[8] Mendoza J.L., Schmidt A., Li Q., Nuvaga E., Barrett T., Bridges R.J., Feranchak A.P., Brautigam C.A., Thomas P.J. (2012). Requirements for efficient correction of ΔF508 CFTR revealed by analyses of evolved sequences. Cell.

[9] Jensen T.J., Loo M.A., Pind S., Williams D.B., Goldberg A.L., Riordan J.R. (1995). Multiple Proteolytic Systems, Including the Proteasome, Contribute to Cftr Processing. Cell.

[10] Ward C.L., Omura S., Kopito R.R. (1995). Degradation of CFTR by the ubiquitin-proteasome pathway. Cell.

[11] Meacham G.C., Patterson C., Zhang W., Younger J.M., Cyr D.M. (2001). The Hsc70 co-chaperone CHIP targets immature CFTR for proteasomal degradation. Nat. Cell Biol..

[12] Younger J.M., Chen L., Ren H.Y., Rosser M.F., Turnbull E.L., Fan C.Y., Patterson C., Cyr D.M. (2006). Sequential quality-control checkpoints triage misfolded cystic fibrosis transmembrane conductance regulator. Cell.

[13] El Khouri E., Le Pavec G., Toledano M.B., Delaunay-Moisan A. (2013). RNF185 is a novel E3 ligase of endoplasmic reticulum-associated degradation (ERAD) that targets cystic fibrosis transmembrane conductance regulator (CFTR). J. Biol. Chem..

[14] Morito D., Hirao K., Oda Y., Hosokawa N., Tokunaga F., Cyr D.M., Tanaka K., Iwai K., Nagata K., Sommer K. (2008). Gp78 cooperates with RMA1 in endoplasmic reticulum-associated degradation of CFTR Delta F508. Mol. Biol. Cell..

[15] Van de Weijer M.L., Krshnan L., Liberatori S., Guerrero E.N., Robson-Tull J., Hahn L., Jan Lebbink R., Wiertz E.J.H.J., Fischer R., Ebner D. (2020). Quality Control of ER Membrane Proteins by the RNF185/Membralin Ubiquitin Ligase Complex. Mol. Cell.

[16] Crosas B., Hanna J., Kirkpatrick D.S., Zhang D.P., Tone Y., Hathaway N.A., Buecker C., Leggett D.S., Schmidt M., King R.W. (2006). Ubiquitin chains are remodeled at the proteasome by opposing ubiquitin ligase and deubiquitinating activities. Cell.

[17] Fang N.N., Ng A.H., Measday V., Mayor T. (2011). Hul5 HECT ubiquitin ligase plays a major role in the ubiquitylation and turnover of cytosolic misfolded proteins. Nat. Cell Biol..

[18] Sharma M., Pampinella F., Nemes C., Benharouga M., So J., Du K., Bache K.G., Papsin B., Zerangue N., Stenmark H. (2004). Misfolding diverts CFTR from recycling to degradation: Quality control at early endosomes. J. Cell Biol..

[19] Okiyoneda T., Barrière H., Bagdány M., Rabeh W.M., Du K., Höhfeld J., Young J.C., Lukacs G.L. (2010). Peripheral protein quality control removes unfolded CFTR from the plasma membrane. Science.

[20] Denning G.M., Anderson M.P., Amara J.F., Marshall J., Smith A.E., Welsh M.J. (1992). Processing of mutant cystic fibrosis transmembrane conductance regulator is temperature-sensitive. Nature.

[21] Van Goor F., Hadida S., Grootenhuis P.D., Burton B., Stack J.H., Straley K.S., Decker C.J., Miller M., McCartney J., Olson E.R. (2011). Correction of the F508del-CFTR protein processing defect in vitro by the investigational drug VX-809. Proc. Natl. Acad. Sci. USA.

[22] Okiyoneda T., Veit G., Sakai R., Aki M., Fujihara T., Higashi M., Susuki-Miyata S., Miyata M., Fukuda N., Yoshida A. (2018). Chaperone-Independent Peripheral Quality Control of CFTR by RFFL E3 Ligase. Dev. Cell.

[23] Taniguchi S., Ito Y., Kiritani H., Maruo A., Sakai R., Ono Y., Fukuda R., Okiyoneda T. (2022). The Ubiquitin Ligase RNF34 Participates in the Peripheral Quality Control of CFTR (RNF34 Role in CFTR PeriQC). Front. Mol. Biosci..

[24] Kamada Y., Ohnishi Y., Nakashima C., Fujii A., Terakawa M., Hamano I., Nakayamada U., Katoh S., Hirata N., Tateishi H. (2023). HERC3 E3 ligase provides an ERAD branch eliminating select membrane proteins. bioRxiv..

[25] Taniguchi S., Ono Y., Doi Y., Taniguchi S., Matsuura Y., Iwasaki A., Hirata N., Fukuda R., Inoue K., Yamaguchi M. (2023). Identification of α-Tocopherol succinate as an RFFL-substrate interaction inhibitor inducing peripheral CFTR stabilization and apoptosis. Biochem. Pharmacol..

[26] Veit G., Avramescu R.G., Perdomo D., Phuan P.W., Bagdany M., Apaja P.M., Borot F., Szollosi D., Wu Y.-S., Finkbeiner W.E. (2014). Some gating potentiators, including VX-770, diminish Delta F508-CFTR functional expression. Sci. Transl. Med..

[27] Kamada Y., Fukuda R., Okiyoneda T. (2019). ELISA Based Protein Ubiquitylation Measurement. Bio-Protoc..

[28] Keating D., Marigowda G., Burr L., Daines C., Mall M.A., McKone E.F., Ramsey B.W., Rowe S.M., Sass L.A., Tullis E. (2018). VX-445-Tezacaftor-Ivacaftor in Patients with Cystic Fibrosis and One or Two Phe508del Alleles. N. Engl. J. Med..

[29] Du K., Lukacs G.L. (2009). Cooperative assembly and misfolding of CFTR domains in vivo. Mol. Biol. Cell.

[30] Chu B.W., Kovary K.M., Guillaume J., Chen L.C., Teruel M.N., Wandless T.J. (2013). The E3 ubiquitin ligase UBE3C enhances proteasome processivity by ubiquitinating partially proteolyzed substrates. J. Biol. Chem..

[31] Yoshida Y., Saeki Y., Murakami A., Kawawaki J., Tsuchiya H., Yoshihara H., Shindo M., Tanaka K. (2015). A comprehensive method for detecting ubiquitinated substrates using TR-TUBE. Proc. Natl. Acad. Sci. USA.

[32] Tsuchiya H., Burana D., Ohtake F., Arai N., Kaiho A., Komada M., Tanaka K., Saeki Y. (2018). Ub-ProT reveals global length and composition of protein ubiquitylation in cells. Nat. Commun..

[33] Zhang D., Raasi S., Fushman D. (2008). Affinity makes the difference: Nonselective interaction of the UBA domain of Ubiquilin-1 with monomeric ubiquitin and polyubiquitin chains. J. Mol. Biol..

[34] Raasi S., Varadan R., Fushman D., Pickart C.M. (2005). Diverse polyubiquitin interaction properties of ubiquitin-associated domains. Nat. Struct. Mol. Biol..

[35] Ritorto M.S., Ewan R., Perez-Oliva A.B., Knebel A., Buhrlage S.J., Wightman M., Kelly S.M., Wood N.T., Virdee S., Gray N.S. (2014). Screening of DUB activity and specificity by MALDI-TOF mass spectrometry. Nat. Commun..

[36] Hospenthal M.K., Mevissen T.E.T., Komander D. (2015). Deubiquitinase-based analysis of ubiquitin chain architecture using Ubiquitin Chain Restriction (UbiCRest). Nat. Protoc..

[37] Brodsky J.L., Wojcikiewicz R.J. (2009). Substrate-specific mediators of ER associated degradation (ERAD). Curr. Opin. Cell Biol..

[38] Vashist S., Ng D.T. (2004). Misfolded proteins are sorted by a sequential checkpoint mechanism of ER quality control. J. Cell Biol..

[39] Leto D.E., Morgens D.W., Zhang L.C., Walczak C.P., Elias J.E., Bassik M.C., Kopito R.R. (2019). Genome-wide CRISPR Analysis Identifies Substrate-Specific Conjugation Modules in ER-Associated Degradation. Mol. Cell.

[40] Hoof T., Demmer A., Hadam M.R., Riordan J.R., Tümmler B. (1994). Cystic fibrosis-type mutational analysis in the ATP-binding cassette transporter signature of human P-glycoprotein MDR1. J. Biol. Chem..

[41] Loo T.W., Clarke D.M. (1997). Correction of defective protein kinesis of human P-glycoprotein mutants by substrates and modulators. J. Biol. Chem..

[42] Chen Y.H., Huang T.Y., Lin Y.T., Lin S.Y., Li W.H., Hsiao H.J., Yan R.L., Tang H.W., Shen Z.Q., Chen G.C. (2021). VPS34 K29/K48 branched ubiquitination governed by UBE3C and TRABID regulates autophagy, proteostasis and liver metabolism. Nat. Commun..

[43] Capurro V., Tomati V., Sondo E., Renda M., Borrelli A., Pastorino C., Guidone D., Venturini A., Giraudo A., Bertozzi S.M. (2021). Partial Rescue of F508del-CFTR Stability and Trafficking Defects by Double Corrector Treatment. Int. J. Mol. Sci..

[44] Haardt M., Benharouga M., Lechardeur D., Kartner N., Lukacs G.L. (1999). C-terminal truncations destabilize the cystic fibrosis transmembrane conductance regulator without impairing its biogenesis. A Nov. Cl. Mutat. J. Biol. Chem..

[45] Cruz C., Ventura F., Bartrons R., Rosa J.L. (2001). HERC3 binding to and regulation by ubiquitin. Febs Lett..

[46] Fiedorczuk K., Chen J. (2022). Molecular structures reveal synergistic rescue of Δ508 CFTR by Trikafta modulators. Science.

[47] Fiedorczuk K., Chen J. (2022). Mechanism of CFTR correction by type I folding correctors. Cell.

[48] You J., Pickart C.M. (2001). A HECT domain E3 enzyme assembles novel polyubiquitin chains. J. Biol. Chem..

[49] Michel M.A., Elliott P.R., Swatek K.N., Simicek M., Pruneda J.N., Wagstaff J.L., Freund S.M., Komander D. (2015). Assembly and specific recognition of k29- and k33-linked polyubiquitin. Mol. Cell.

[50] Gottlieb C.D., Thompson A.C.S., Ordureau A., Harper J.W., Kopito R.R. (2019). Acute unfolding of a single protein immediately stimulates recruitment of ubiquitin protein ligase E3C (UBE3C) to 26S proteasomes. J. Biol. Chem..

[51] Jariel-Encontre I., Bossis G., Piechaczyk M. (2008). Ubiquitin-independent degradation of proteins by the proteasome. Biochim. Biophys. Acta.

[52] Palicharla V.R., Gupta D., Bhattacharya D., Maddika S. (2021). Ubiquitin-independent proteasomal degradation of Spindlin-1 by the E3 ligase HACE1 contributes to cell-cell adhesion. FEBS Lett..

[53] Yuan B., Liu J., Shi A., Cao J., Yu Y., Zhu Y., Zhang C., Qiu Y., Luo H., Shi J. (2023). HERC3 promotes YAP/TAZ stability and tumorigenesis independently of its ubiquitin ligase activity. EMBO J..

[54] Hjerpe R., Aillet F., Lopitz-Otsoa F., Lang V., England P., Rodriguez M.S. (2009). Efficient protection and isolation of ubiquitylated proteins using tandem ubiquitin-binding entities. EMBO Rep..

[55] Sims J.J., Scavone F., Cooper E.M., Kane L.A., Youle R.J., Boeke J.D., Cohen R.E. (2012). Polyubiquitin-sensor proteins reveal localization and linkage-type dependence of cellular ubiquitin signaling. Nat. Methods.

[56] Kristariyanto Y.A., Abdul Rehman S.A., Campbell D.G., Morrice N.A., Johnson C., Toth R., Kulathu Y. (2015). K29-selective ubiquitin binding domain reveals structural basis of specificity and heterotypic nature of k29 polyubiquitin. Mol. Cell.

[57] Gentzsch M., Chang X.B., Cui L., Wu Y., Ozols V.V., Choudhury A., Pagano R.E., Riordan J.R. (2004). Endocytic trafficking routes of wild type and DeltaF508 cystic fibrosis transmembrane conductance regulator. Mol. Biol. Cell..

